# Hierarchical Neutral and Non‐Neutral Spatial Genetic Structuring in the European Sardine (*Sardina pilchardus*) Revealed by Genomic Analysis: Implications for Management

**DOI:** 10.1111/eva.70080

**Published:** 2025-04-01

**Authors:** Niall J. McKeown, Christophe Lebigre, Jeroen van der Kooij, Martin Huret

**Affiliations:** ^1^ Department of Life Sciences Aberystwyth University Aberystwyth Ceredigion, Wales UK; ^2^ DECOD (ecosystems and sustainability), Institut Agro, IFREMER INRAE Plouzané France; ^3^ CEFAS Lowestoft Suffolk UK

**Keywords:** adaptation, fishery, genetic, small pelagic, stock, sustainability

## Abstract

The European sardine ( *Sardina pilchardus* ) sustains some of the most important East Atlantic fisheries and is exhibiting pronounced phenotypic and distributional changes linked to environmental changes. The application of high‐resolution genomic methods is recommended to provide insights into population demographics and patterns of ecological and evolutionary diversification. This study performed genome wide SNP analysis of samples collected across understudied NE Atlantic waters as well as geographical outgroup samples from Morocco and the Western Mediterranean. The data revealed pronounced differentiation of three regional groups (NE Atlantic, Morocco, and Western Mediterranean) that can be linked to glacial vicariance and contemporary dispersal limitations. Structuring was also apparent at outlier loci adding to evidence that genome architecture and non‐neutral processes are influencing sardine populations at various spatial scales. The highly resolved Morocco group may be a previously undescribed and localized lineage and confirms complex stock structure along the North African coast. Among the NE Atlantic samples, genome wide patterns confirm restricted gene flow between Biscay and North Sea sardine with signatures of isolation by distance. *F*_ST_, individual assignment, and introgression tail analyses of outlier loci revealed further structuring and identify a North Sea—Eastern Channel group distinct from a Bay of Biscay‐Celtic Sea‐Western Channel group. This pattern contradicts current management boundaries and indicates that increasing sardine numbers in the North Sea reflect an expansion of an eastern English Channel‐North Sea fringe population. While this confirms the ability of the species' northern peripheral populations to expand in response to changing conditions, the genetically differentiated southern populations may differ in this regard. Overall, this study adds to a developing genetic framework for understanding sardine biocomplexity and provides resources for management.

## 1 Introduction

Small pelagic fish provide approximately 30% of the world's annual fish harvest, supporting artisanal and major industrial fisheries across the globe (Freon et al. 2005; FAO 2022). They respond quickly to environmental changes, explaining their marked fluctuations in abundance as well as the rapid extension of their distribution (Peck et al. 2013). They also represent a key component of marine ecosystems, serving as a trophic link between plankton and predators (Cury et al. 2000). Their dramatic stock fluctuations have often had dire economic consequences for fishing communities, and profoundly impacted the functioning of ecosystems (Alheit et al. 2012). Accordingly, the integration of accurate information of population structure and demographics of small pelagic fish is widely recognized as an essential step to ensure fishery sustainability, broader ecosystem functioning, and future food security (Reiss et al. 2009; Kerr et al. 2017).

The European sardine ( *Sardina pilchardus* ) is a small pelagic schooling fish distributed in coastal waters of the eastern Atlantic Ocean (from Western Africa to the North Sea) and in the Mediterranean and Black Seas (Grant and Bowen 1998). This species has spawning grounds throughout its distribution, has a high dispersal potential (Garrido et al. 2016, 2017), and exhibits pronounced variation in life‐history traits and/or abundance at both larval and juvenile/adult stages linked to climatic variability (Silva et al. 2008, 2009; Garrido et al. 2016, 2017; Menu et al. 2023). Sardine abundance is reported to have increased in the North Sea since the 1990s (Alheit et al. 2012) with this expansion linked to climate variability (Alheit et al. 2012) and paralleling recent northward expansions in European waters reported for other Lusitanian pelagic species (e.g., Van der Kooij et al. 2024; Montero‐Serra et al. 2015).

Sardine is heavily exploited throughout much of its range and represents one of the most valued fisheries in the East Atlantic (FAO 2022; ICES 2022). In European Atlantic waters, three stocks are considered in its management: a Northern stock (Celtic Sea to English Channel, ICES area VII), a Central Stock (Bay of Biscay, ICES sub‐areas VIIIa‐VIIIb‐VIIId, encompassing statistical rectangles 25E4 and 25E5 of the area VII at the tip of Brittany, France), and a Southern Stock (spanning the Cantabrian Sea to the gulf of Cadiz sub‐areas VIIIc‐IXa; ICES 2022). A further three stocks are described along the Moroccan coast (FAO 2022), with an even greater number of stocks in the Mediterranean Sea (Neves et al. 2021, 2023). However, the validity of current management boundaries is a matter of debate because there are clear discrepancies between sardine operational stock definitions and the species' spatial structuring in key biological and population characteristics (reviewed in Caballero‐Huertas et al. 2022). Attempts to address uncertainties in sardine population structure is timely as catches in many regions have declined and several stocks are recognized as fully exploited (ICES 2022). However, the numerous phenotypic studies based on body morphometrics (Baibai et al. 2012; Mounir et al. 2019), otolith shape and microchemistry (Correia et al. 2014; Jemaa et al. 2015; Neves et al. 2021, 2023), and genetic studies based on allozymes (Chlaida et al. 2006, 2009; Laurent and Planes 2007; Laurent et al. 2007), mtDNA (Tinti et al. 2002; Atarhouch et al. 2006), and microsatellites (Gonzalez and Zardoya 2007; Ruggeri et al. 2012, 2013; Kasapidis et al. 2012) have generally provided inconclusive, and at times contradictory, delineation of population boundaries (Neves et al. 2021). A central issue is that genetic studies, that enable us to directly test restricted interbreeding, have typically reported weak or absent population structure over wide geographical areas (Caballero‐Huertas et al. 2022). While such a lack of structure would be compatible with the species' dispersal potential, it may hide a diversity of scenarios with regard to the demo‐genetic significance of phenotypic differences revealed in some studies (Neves et al. 2023).

Modern genomic approaches are providing unprecedented sensitivity for resolving patterns of population structure, individual dispersal, and local adaptation in marine species with direct application to sustainable fishery management (Ahrens et al. 2018; Mullins et al. 2018; McKeown et al. 2020). Recent genome wide studies by Antoniou et al. (2023) and da Fonseca et al. (2024) have reported robust genetic divergence between Mediterranean and Atlantic sardine for the first time. da Fonseca et al. (2024) also reported, within Atlantic waters, deep divergence between a western group (Azores and Madeira) and central group (Iberian Peninsula and Bay of Biscay). There is the recognized need to leverage the power of such genomic analyses to understand sardine populations within the species less well studied northern range to resolve stock boundaries and understand the genetic processes underpinning observed distribution/abundance changes linked to climate change in such areas (McKeown et al. 2024). In the only genomic study of sardine from such northern waters (Bay of Biscay to North Sea) to date, McKeown et al. (2024) did identify a small number of outlier loci (*n* = 14) that revealed a significant correlation between *F*_ST_ and geographical distance. While these results suggest that sardine is not panmictic throughout the Biscay‐North Sea region, the limited sampling (e.g., inclusion of only a single North Sea site) and low level of genome‐wide and outlier divergence provided limited insight into the roles of selection and dispersal limitations, and demographic structuring in relation to management units. Accordingly, the primary objective of this study was to analyze population structure in the currently understudied NE Atlantic waters spanning the Bay of Biscay, the Celtic Sea, the English Channel, and the North Sea, employing a greater number of loci and a substantially more comprehensive sampling regime than McKeown et al. (2024). This not only enabled us to measure the genetic structure across the current central (Biscay) and northern (Celtic Sea and English Channel) stocks but also to assess the genetic composition in a leading‐edge (North Sea) population and investigate temporal changes in levels of variation. Importantly, we also included geographic outgroup samples from Morocco and the western Mediterranean. These permitted our results to be linked to genomic studies of sardine in more southern waters (Antoniou et al. 2023; da Fonseca et al. 2024), to provide a much needed macrogeographical context.

## 2 Materials and Methods

### 2.1 Sample Collection and DNA Extraction

**FIGURE 1:**
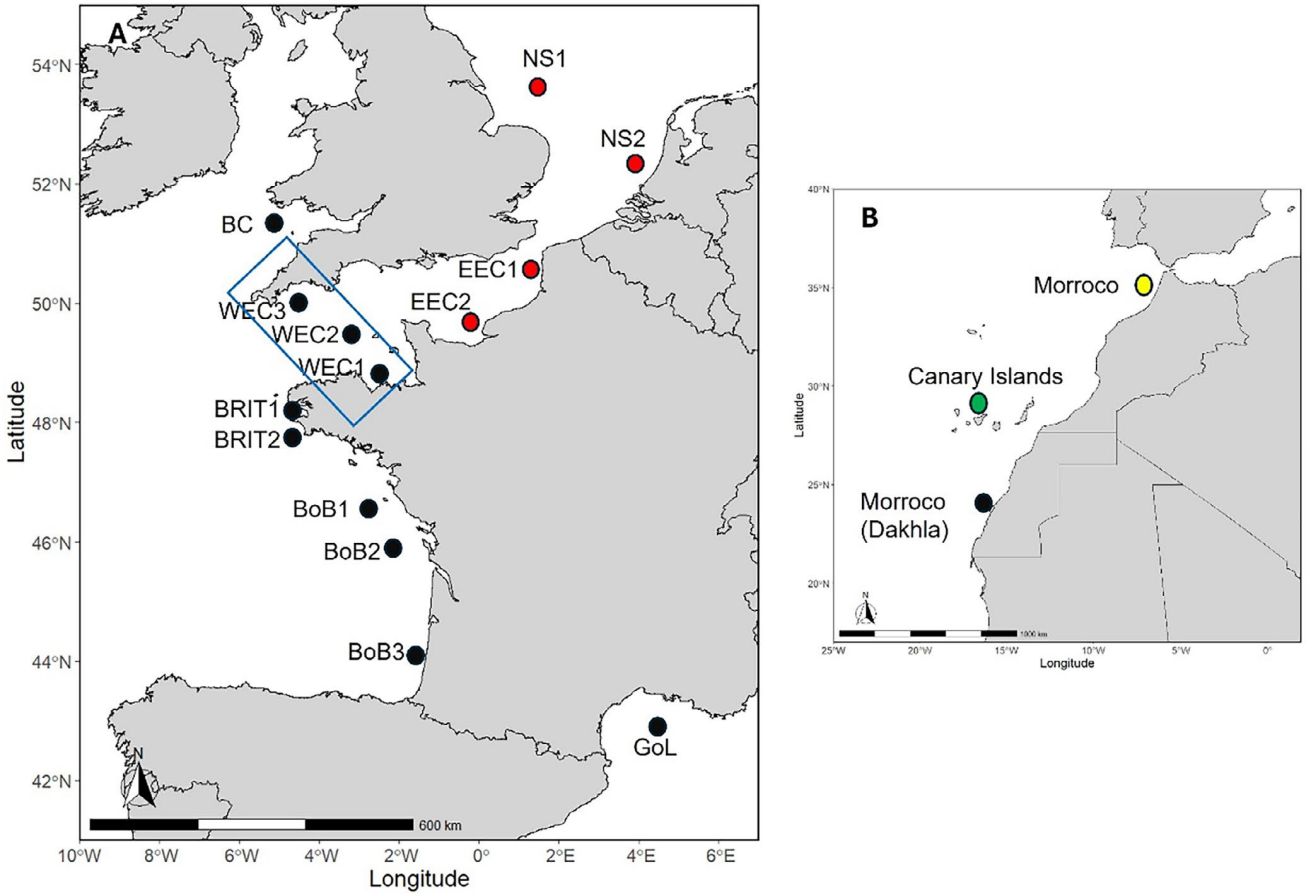
(A) Map showing the location of the collected samples in the NE Atlantic and Western Mediterranean. Sample sites for which sardine exhibited signs of membership to an East Channel‐North Sea group are denoted in red. The blue rectangle highlights the Western channel samples for which a flip in the relative proportion of southern group ancestry were revealed by spatial gradient analysis. (B) Location of the Dakhla sample collected here in relation to the sample sites in the Canary Islands (green disc) and Northern Morocco included in the study by da Fonseca et al. (2024).

Samples from the Bay of Biscay (Garren et al. 2019), Celtic Sea at the entrance of the Bristol Channel (Garren et al. 2019), English Channel (van der Kooij 2020), North Sea (Lazard and Auber 2020), and the Gulf of Lion (Bourdeix 2020) within the Western Mediterranean Sea, were collected during research surveys as well as from commercial fishing catches, while samples from a Moroccan site was provided by the French canning industry. The data collection followed the required legislations of the countries in which fish were captured. Sample sites are depicted in Figure 1 and details are provided in Table 1. In all cases, samples comprised mixed age and size classes of adults except for the sample WEC2 which comprised post‐larvae. DNA was extracted from ethanol‐preserved tissue samples using a Qiagen DNeasy Blood and Tissue kit (Qiagen, GmbH, Hilden, Germany) following manufacturer's instructions.

**TABLE 1:** Sample information and summary indices of genetic variation including number of polymorphic loci (Npoly), observed and expected heterozygosities (*H*_O_ and *H*_E_, respectively), and *F*_IS_ value (all values nonsignificant).

Region	Code name	Lat (°N)/long (°W)	Sample date (DD‐MM‐YYYY)	Number	Total SNPs (*n* = 3592)				Outlier SNPs (*n* = 223)			
					Npoly	*H*_O_	*H*_E_	*F*_IS_	Npoly	*H*_O_	*H*_E_	*F*_IS_
North Sea	NS1	54.191/1.356	23‐01‐2020	19	3420	0.242	0.262	0.080	158	0.304	0.335	0.091
North Sea	NS2	52.253/3.914	15‐01‐2020	18	3404	0.238	0.259	0.085	78	0.323	0.337	0.454
East English Channel	EEC1	50.481/1.445	23‐01‐2020	14	3216	0.253	0.275	0.083	98	0.348	0.361	0.414
East English Channel	EEC2	49.633/−0.007	26‐06‐2020	21	3453	0.236	0.259	0.095	197	0.271	0.332	0.183
West English Channel	WEC1	48.7/−2.5	24‐10‐2019	13	3239	0.248	0.275	0.104	130	0.273	0.34	0.205
West English Channel	WEC2	49.5/−3.0	10‐10‐2020	10	2847	0.267	0.299	0.112	40	0.328	0.357	0.079
West English Channel	WEC3	50.0/−4.6	16‐10‐2020	19	3432	0.247	0.262	0.059	161	0.317	0.349	0.094
Bristol Channel	BC	51.248/−5.498	03‐12‐2019	19	3451	0.243	0.265	0.083	148	0.301	0.363	0.175
Brittany	BRIT1	48.15/−4.4	25‐05‐2020	20	3480	0.247	0.263	0.058	175	0.385	0.371	0.031
Brittany	BRIT2	47.9/−4.6	25‐05‐2020	19	3475	0.247	0.263	0.062	158	0.362	0.385	0.064
Bay of Biscay	BoB1	46.592/−2.605	01‐11‐2019	20	3472	0.243	0.261	0.071	111	0.351	0.382	0.084
Bay of Biscay	BoB2	46.054/−2.056	31‐10‐2019	15	3339	0.247	0.272	0.090	138	0.319	0.357	0.108
Bay of Biscay	BoB3	44.061/−1.391	29‐10‐2019	18	3445	0.243	0.267	0.096	160	0.309	0.373	0.175
Morocco (Dakhla)	MOR	23.85/−16.43	01‐01‐2020	20	3270	0.243	0.264	0.081	81	0.212	0.262	0.186
Western Mediterranean Sea	GoL	43.10/4.20	21‐09‐2020	24	3439	0.243	0.257	0.057	207	0.277	0.301	0.083

*Note:* Genetic indices are reported for the entire SNP dataset (*n* SNP = 3592) and for the outlier SNPs identified across all samples (*n* SNP = 223).

### 2.2 Restriction Enzyme‐Associated DNA Sequencing (RAD‐Seq) and Genotyping

Genome wide SNP analyses were performed using tuneable genotyping by sequencing (tGBS) (Ott et al. 2017) of a Bsp1286I digested library sequenced on an Illumina HiSeq X (Illumnina Inc., San Diego, CA, USA). Sequenced reads were analyzed using a custom Perl script (available at https://github.com/orgs/schnablelab), which assigned each read to a sample and removed barcode sequences. “Seqclean” (https://sourceforge.net/projects/seqclean) was used to remove adaptor sequences and chimeric reads harboring internal restriction enzyme sites. Retained reads were subjected to quality trimming in two phases using the software Lucy2 (Li and Chou 2004) in which bases with PHRED scores < 20 (of 40) were removed. In the first phase, sequences were scanned at each end; in the second phase, sequences were scanned using overlapping 10 bp windows. Quality trimmed sequence reads were aligned to a reference genome (Genbank accession: GCA_900499035.1) using GSNAP (Wu and Nacu 2010) and only reads with a single unique alignment were retained for subsequent analysis. For the retained reads, a SNP was called homozygous in an individual if at least 25 reads supported the genotype at the site and at least 90% of all reads covering that site shared the same nucleotide. A SNP was considered heterozygous in an individual if each of the two nucleotide variants were reported at least 15 times, and each allele was represented in more than 35% of the total reads. Polymorphisms in the first and last 3 bp of each sequence were ignored. To reduce biases that may be introduced by retaining low frequency SNPs (Roesti et al. 2012), the minimum allele frequency (MAF) was set at 5%.

### 2.3 Summary Statistics and Analysis of Genetic Structure

Allele frequencies and observed (*H*_O_) and expected (*H*_E_) heterozygosities were estimated using ARLEQUIN 3.4.2.2 (Excoffier and Lischer 2010). ARLEQUIN was also used to test for departures from expectations of Hardy–Weinberg Equilibrium (HWE). Genetic differentiation among samples was quantified by global and pairwise *F*_ST_ (Weir and Cockerham 1984) with statistical significances evaluated in ARLEQUIN with 10,000 permutations and a missing data threshold of 0.1 per locus. Mantel tests, implemented in GENALEX (Peakall and Smouse 2006), were used to test the relationship between genetic distances (pairwise *F*_ST_) and geographical distances between sample sites (i.e., isolation by distance). Geographical distances were calculated along a least cost path between each sampling location using the r‐package “MarMap.” GENALEX was also used to perform a principal co‐ordinate analysis of *F*_ST_ between samples. The Bayesian clustering method implemented in STRUCTURE 2.3.4 (Pritchard et al. 2000) was employed to (i) identify the most probable number of genetically distinct groups (*K*) represented by the data and (ii) estimate assignment probabilities (*Q*) for each individual (specifically their genomic components) to these groups. The analysis was performed with and without the LOCPRIOR model, in both cases assuming admixture. Simulations were run 10 times for each proposed *K* (1–5; higher values of *K* were tested in shorter pilot runs) to assess convergence. Each run had a burn‐in of 100,000 Markov Chain Monte Carlo (MCMC) samples followed by 1,000,000 MCMC repetitions. Models were assessed using *L*(*K*) (Pritchard et al. 2000) and Δ*K* (Evanno et al. 2005). To complement the STRUCTURE analysis, individual assignment (IA) tests were performed in GENECLASS 2 (Piry et al. 2004). These tests included (i) assignment of individuals treated as unknown to defined reference groups and (ii) self‐classification tests to defined reference groups, with the various reference groups configured according to results from *F*_ST_ and STRUCTURE analyses.

### 2.4 Detection of Outliers and Analysis of Introgression Gradients

Outlier loci (i.e., exhibiting divergence beyond neutral expectations) were identified using the independent approaches implemented in BAYESCAN 2.0 (Foll and Gaggiotti 2008) and the hierarchical FDIST test in ARLEQUIN. For the BAYESCAN analysis, all parameters that could be modified were left as default and the false discovery rate was set at 5% meaning that a marker with a *q* value lower than 0.05 was considered an outlier. In the FDIST analysis, loci with significantly higher *F*_ST_ values (*p* < 0.05) were considered outliers. Only SNPs identified as non‐neutral by both BAYESCAN and FDIST methods were retained as outliers for subsequent analyses. The stringent parameters and consensus approach to outlier identification was employed to account for potential false positives (Narum and Hess 2011; Ahrens et al. 2018). The outlier tests were performed globally (i.e., across all samples) and between pairs of samples as recommended by Vitalis et al. (2001). The functional significance of outlier loci was investigated by analyzing the SNP containing sequences using BLAST following Milano et al. (2014).

Outlier loci have been shown to be powerful tools to identify barriers to gene flow via introgression tail analysis (Robinet et al. 2020). Gene flow between differentiated lineages can generate dynamic spatial gradients in admixture between lineages. Breaks in such ancestry gradients can indicate local reductions in the spread of foreign alleles and reveal barriers to dispersal that may not be strong enough to produce genetic differentiation (significant *F*_ST_ values) at migration‐drift equilibrium (Gagnaire et al. 2015). As outlier loci may be more resistant to introgression than neutral loci, they may maintain their ancestral identity for longer, and thus provide a signature of such barriers (Gagnaire et al. 2015). Robinet et al. (2020) employed spatial gradient analysis of outlier loci to assess Mediterranean ancestry levels among seabass to identify biologically plausible dispersal barriers in the NE Atlantic. As our study identified outliers differentiating three regional groups (Mediterranean, Morocco, and NE Atlantic), we employed a similar introgression tail analysis as Robinet et al. (2020). Specifically, we inspected the spatial gradient in Mediterranean and Moroccan ancestry levels among the NE Atlantic samples to test for local dispersal barriers that could be revealed by shifts or breaks in the ancestry gradients. The proportions of Mediterranean and Moroccan ancestry among the NE Atlantic samples were inferred using the program Admixture (Alexander et al. 2009) wherein *K* was set to 3 with the Mediterranean and Moroccan samples included as references and the termination criterion was set to 100 iterations.

## 3 Results

### 3.1 Genome Wide Diversity

For the initial sample of 288 individuals, a total of 2× 659,570,156 sequence reads were obtained with an average of 2× 2,290,174 per individual (average read length = 143 bp; minimum read length = 30 bp; maximum read length = 211 bp). Following trimming to 142 bp and exclusion of sequences that aligned to > 1 location in the genome a total of 98,906 SNPs were identified and genotyped in at least 50% of individuals. Nineteen individuals were removed at this stage due to high missing data rates. The remaining genotypes were further filtered to retain only SNPs that were genotyped in at least 90% of individuals. This resulted in a final dataset comprising 269 individuals and 3592 biallelic SNPs which were used for downstream analysis.

### 3.2 Regional Genetic Structure

**FIGURE 2:**
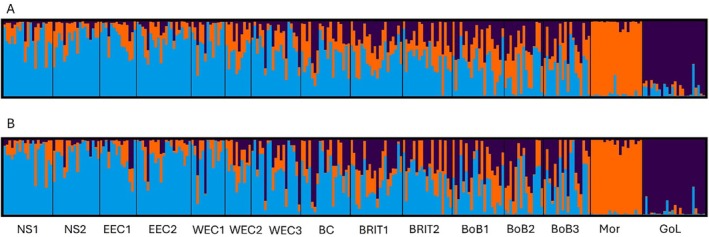
STRUCTURE bar plots showing the clustering of individuals based on (A) the entire SNP dataset (3592 SNPs) and (B) the outlier SNP dataset (223 SNPs). Results are for *K* = 3 which was the optimal model in all cases.

Based on the entire SNP dataset, all samples exhibited similar levels of multilocus variability and conformance to HWE equilibrium (Table 1). Genetic differentiation across all samples was significant (global *F*_ST_ = 0.019; *p* < 0.001) and pairwise *F*_ST_ clearly indicated that this overall genetic structure was largely attributable to Moroccan and Mediterranean samples. These samples were significantly differentiated from each other (*F*_ST_ = 0.119; *p* < 0.001) and to all other samples (Bay of Biscay, English Channel, Bristol Channel, and North Sea; hereafter referred to as NE Atlantic samples; Table 2). Pairwise comparison between the Morocco and Mediterranean samples to NE Atlantic samples yielded similar average pairwise *F*_ST_ of 0.046 and 0.047, respectively (Table 2). This regional structure was resolved by the STRUCTURE clustering analysis which reported an optimal model of *K* = 3 wherein the Moroccan and Mediterranean individuals each assigned to their own discrete clusters with the NE Atlantic samples all assigning to one cluster (Figure 2A).

**TABLE 2:** *F*_ST_ values between pairs of samples.

	NS1	NS2	EEC1	EEC2	WEC1	WEC2	WEC3	BC	BRIT1	BRIT2	BoB1	BoB2	BoB3	Mor	GoL
NS1	—	0.002	−0.004	0.012	0.013	0.014	**0.026**	**0.041**	**0.046**	**0.048**	**0.062**	**0.109**	**0.069**	**0.359**	**0.368**
NS2	−0.003	—	0.005	0.020	**0.020**	**0.032**	**0.031**	**0.045**	**0.043**	**0.036**	**0.072**	**0.110**	**0.076**	**0.392**	**0.357**
EEC1	−0.006	−0.003	—	0.007	**0.015**	**0.029**	0.005	**0.018**	**0.016**	**0.018**	**0.047**	**0.096**	**0.042**	**0.380**	**0.332**
EEC2	−0.000	−0.002	−0.002	—	0.002	**0.045**	**0.023**	**0.042**	**0.044**	**0.047**	**0.061**	**0.114**	**0.075**	**0.371**	**0.349**
WEC1	−0.000	−0.000	0.000	−0.002	—	0.026	0.011	0.022	**0.026**	**0.023**	**0.052**	**0.099**	**0.053**	**0.400**	**0.323**
WEC2	−0.014	−0.003	−0.003	−0.008	−0.001	—	0.008	−0.009	−0.003	−0.006	0.014	**0.042**	−0.001	**0.318**	**0.313**
WEC3	0.002	0.002	−0.002	0.002	0.001	−0.008	—	0.006	0.002	0.013	**0.020**	**0.051**	0.016	**0.361**	**0.268**
BC	0.003	0.002	−0.001	**0.004**	−0.000	−0.016	0.000	—	−0.006	−0.003	0.013	**0.024**	−0.001	**0.313**	**0.263**
BRIT1	**0.006**	0.002	−0.003	**0.006** ^b^	−0.001	−0.018	0.001	−0.001	—	−0.002	0.004	0.012	−0.006	**0.303**	**0.243**
BRIT2	**0.004**	0.002	−0.001	**0.005**	−0.000	−0.015	0.002	−0.001	0.002	—	0.006	**0.027**	0.005	**0.281**	**0.256**
BoB1	**0.006** ^b^	**0.007** ^b^	**0.004**	**0.008** ^b^	**0.005** ^b^	−0.007	0.001	−0.000	0.002	0.002	—	**0.018**	−0.001	**0.274**	**0.247**
BoB2	**0.013** ^b^	**0.012** ^b^	**0.011** ^a^, ^b^	**0.016** ^a^, ^b^	**0.012** ^b^	−0.004	**0.009** ^a^	0.001	0.002	0.003	0.003	—	−0.001	**0.308**	**0.247**
BoB3	**0.009** ^b^	**0.010** ^b^	**0.005**	**0.013** ^a^, ^b^	**0.006**	−0.015	**0.004**	−0.000	**0.004**	0.002	−0.001	−0.000	—	**0.280**	**0.236**
MOR	**0.053** ^a^, ^b^	**0.057** ^a^, ^b^	**0.054** ^a^, ^b^	**0.058** ^a^, ^b^	**0.057** ^a^, ^b^	**0.027** ^a^, ^b^	**0.056** ^a^, ^b^	**0.042** ^a^, ^b^	**0.043** ^a^, ^b^	**0.040** ^a^, ^b^	**0.041** ^a^, ^b^	**0.039** ^a^, ^b^	**0.037** ^a^, ^b^	**—**	**0.589**
GoL	**0.060** ^a^, ^b^	**0.054** ^a^, ^b^	**0.051** ^a^, ^b^	**0.056** ^a^, ^b^	**0.050** ^a^, ^b^	**0.036** ^a^, ^b^	**0.046** ^a^, ^b^	**0.043** ^a^, ^b^	**0.045** ^a^, ^b^	**0.045** ^a^, ^b^	**0.040** ^a^, ^b^	**0.039** ^a^, ^b^	**0.041** ^a^, ^b^	**0.119** ^a^, ^b^	—

*Note:* Lower triangular matrix reports values based on entire SNP dataset; upper triangular matrix reports values derived from the outlier SNPs. Values in bold were statistically significant.

^a^ Denotes comparisons for which the *F*_ST_ calculated excluding the entire suite of outliers (3592 minus 223) was significant, while.

^b^ Denotes those comparisons yielding significant *F*_ST_ following removal of outliers identified among NE Atlantic samples only (3592 minus 38).

**FIGURE 3:**
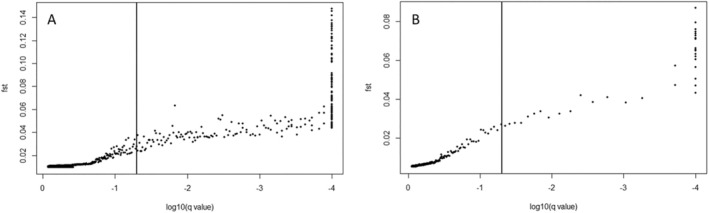
Results of global BAYESCAN analysis across (A) all samples identifying 223 positive outlier SNPs and (B) across the NE Atlantic samples identifying 38 positive outlier SNPs.

Outlier analysis in FDIST identified 598 positive outliers and 223 of these were also identified by BAYESCAN (Figure 3). Outlier analysis using different combination of samples (i.e., excluding different samples) generally reported lower numbers of outliers; but in all cases, these were present among the 223 identified in the global analysis (Table S1). *F*_ST_ values (Table 2) and individual clustering analyses (Figure 2B) of the 223 outliers reported the strong differentiation between Moroccan, Mediterranean, and NE Atlantic. The outlier SNPs had an overall greater level of variation compared to genome wide patterns as well as broadly similar levels of variation among samples except for the Moroccan sample which exhibited reduced numbers of polymorphic loci and heterozygosity levels compared to the other sites. Thirty four of the 223 SNPs yielded significant BLAST results in many cases exhibiting similarity to mRNA sequences from sardine (Table S2). The remaining SNPs containing sequences returned no significant similarity after BLAST analysis. *F*_ST_ and STRUCTURE analyses excluding the 223 outliers (i.e., a presumed neutral dataset of 3369 SNPs) still reported the clear separation between the Moroccan, Mediterranean, and NE Atlantic samples (Table 2; Figure S1).

### 3.3 Spatial Genetic Structure Within the NE Atlantic Group

**FIGURE 4:**
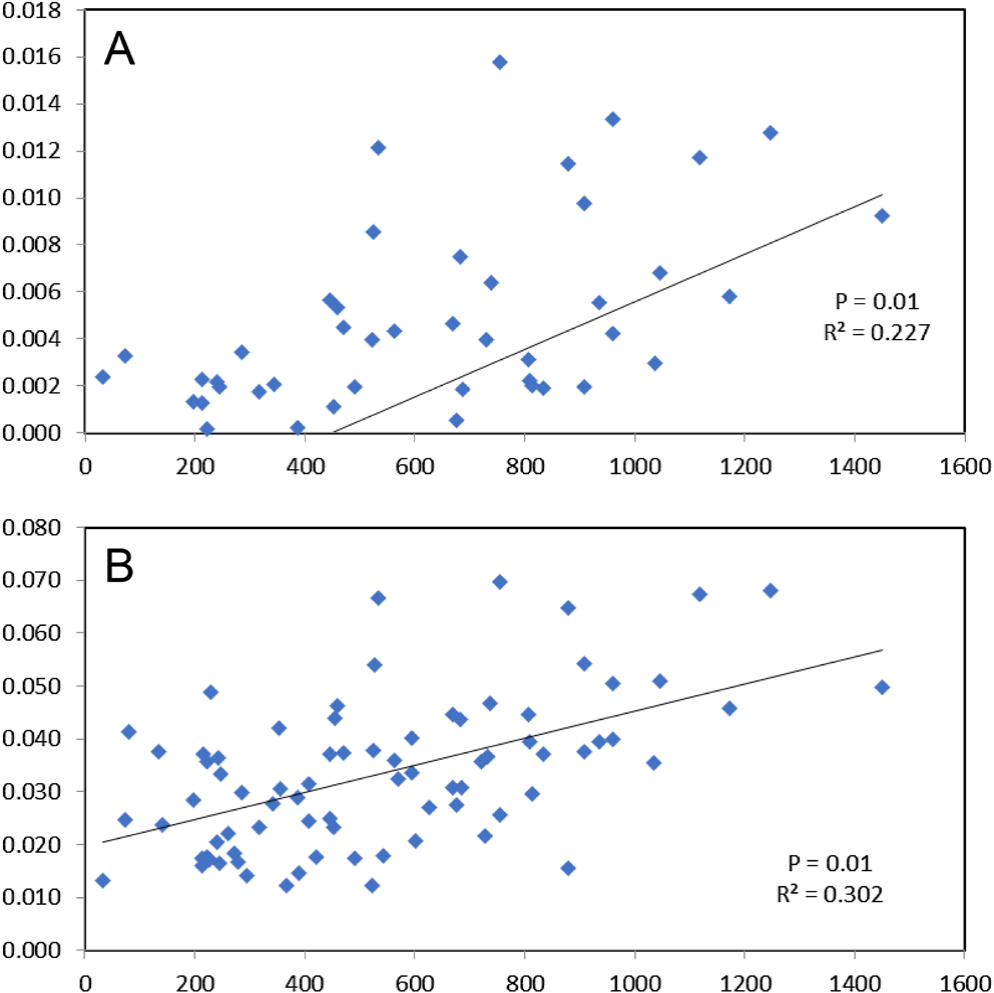
Mantel tests showing the significant correlation between geographical distance (*X* axis) and *F*_ST_ (*Y* axis) based on (A) full SNP dataset (*n* = 3592 SNPs) and (B) outlier SNPs (*n* = 223 SNPs).

Based on the entire SNP dataset, the global *F*_ST_ among the NE Atlantic samples was numerically small and nonsignificant (*F*_ST_ = 0.003; *p* = 0.6). However, there was a spatially coherent pattern of genetic differentiation in pairwise *F*_ST_ values with a significant isolation by distance pattern (Figure 4A). More specifically, the three southern Bay of Biscay samples exhibited significant *F*_ST_ when compared with the two North Sea and two East Channel samples. There was less consistent genetic differentiation of the Biscay samples with the western Channel samples. The western Channel and Bristol Channel samples also exhibited nonsignificant *F*_ST_ in all comparisons with the Brittany samples to the south and the East Channel and North Sea samples.

**FIGURE 5:**
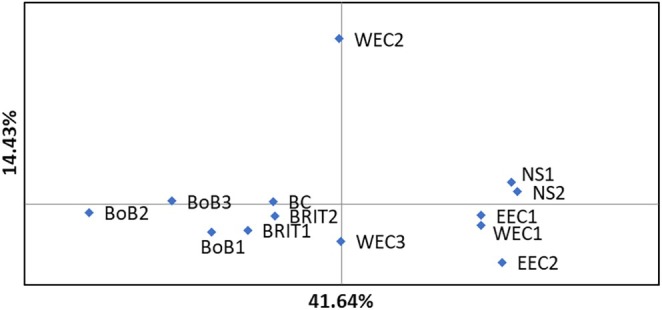
PCoA of NE Atlantic samples based on *F*_ST_ values calculated from the outlier SNPs.

The outlier analysis based on only the NE Atlantic samples (i.e., excluding Morocco and Mediterranean), identified 38 outlier SNPs (Figure 3B), all of which were already identified within the 223 outliers from the global analysis. As both sets of outliers reported near identical patterns, we focus on the results for the full outlier dataset (*n* = 223). Recalculating *F*_ST_ values following removal of global outliers reported a smaller number of significant pairwise comparisons compared to the full SNP dataset; however, significant differentiation between the North Sea/East Channel and Biscay samples was still observed in 10 of 12 comparisons (Table 2). *F*_ST_ analysis of the NE Atlantic samples based on the outlier SNPs revealed a more pronounced correlation with geographical distance (*R* = 0.55) compared to the full SNP dataset (*R* = 0.48; Figure 4A,B). PCoA of *F*_ST_ estimates from the outliers also revealed a clear separation of samples into two groups (Figure 5). The first group consisted of the four samples collected in the most North‐eastern areas (two sampling stations from North Sea, and two sampling stations from Eastern Channel) and a second group comprised the South‐western samples (Bay of Biscay, Brittany, and Bristol Channel). Among the Western English Channel samples, two were clustered with the SW group (WEC2 and WEC3), and one with the NE group (WEC1). The STRUCTURE analysis based on the outliers identified *K* = 1 as the optimal model; however, there was a clear pattern of individual admixture proportions under the *K* = 2 model which was consistent with the separation of North Sea‐East Channel samples from the remaining samples (Table 3). Specifically, the four North Sea‐East Channel samples had mean assignment probabilities of > 0.9 of belonging to one group. In contrast, the Biscay, Brittany, and Bristol Channel samples had much more intermediate probabilities of assigning to either group. The West Channel samples WEC 1 and WEC2 had greater probabilities of assigning to group 1, but these were still lower than the North Sea‐East Channel samples.

**TABLE 3:** Results of assignment tests performed in STRUCTURE and GENECLASS based on 223 outlier SNPs.

Sample name (sample size)	Mean membership proportions		Individual assignment	
	Group 1	Group 2	East Channel‐North Sea group	Biscay—Bristol Channel group
NS1 (18)	0.981	0.019	19	0
NS2 (16)	0.939	0.061	16	2
EEC1 (14)	0.9	0.1	19	2
EEC2 (21)	0.959	0.041	20	1
WEC1 (13)	0.888	0.112	11	2
WEC2 (10)	0.839	0.161	6	4
WEC3 (19)	0.695	0.305	12	7
BC (19)	0.659	0.341	10	9
BRIT1 (20)	0.625	0.375	8	12
BRIT2 (19)	0.641	0.359	9	10
BoB1 (20)	0.564	0.436	9	11
BoB2 (15)	0.449	0.551	4	11
BoB3 (18)	0.528	0.472	7	11

*Note:* STRUCTURE analysis was performed under a model of *K* = 2 with the mean membership proportion referring to the average *Q* values of individuals from each location of belonging to either group. Two types of individual assignment tests were performed. First, based on *F*_ST_ and STRUCTURE results, samples were separated into two groups East Channel‐North Sea group (comprising NS1, NS2, EEC1, and EEC2) and a Biscay to Bristol Channel group (comprising BoB1‐3, BRIT1, BRIT2, and BC) and levels of self‐classification measured using the “Leave one out” option. Second, the West Channel samples (WEC1‐3, shown in dark grey) were treated as unknowns and assigned to the aforementioned reference groups.

Based on the STRUCTURE, *F*_ST_, and geographical sampling locations, various samples were grouped into a northern group (NS1, NS2, EEC1, EEC2) and a southern group (BoB1, BoB2, BoB3, BRIT1, BRIT2, and BC) and self‐classification tests performed using the “leave one out” method and Nei's DA as an assignment distance. These results emphasized the distinctiveness of the northern group with 93% of these individuals being self‐assigned to the northern group (Table 3). Assignment strength was much weaker for the southern group with 58% self‐assignment and 42% of individuals assigning to the northern group (Table 3). Assignment tests were performed using the same north and south reference groups but treating the West Channel samples, which showed geographically discordant patterns based on *F*_ST_ and intermediate assignment probabilities from STRUCTURE, as “unknown.” Assignment of these unknown individuals was skewed toward the Northern group (Table 3).

### 3.4 Spatial Gradients in Admixture Among NE Atlantic Sardine

**FIGURE 6:**
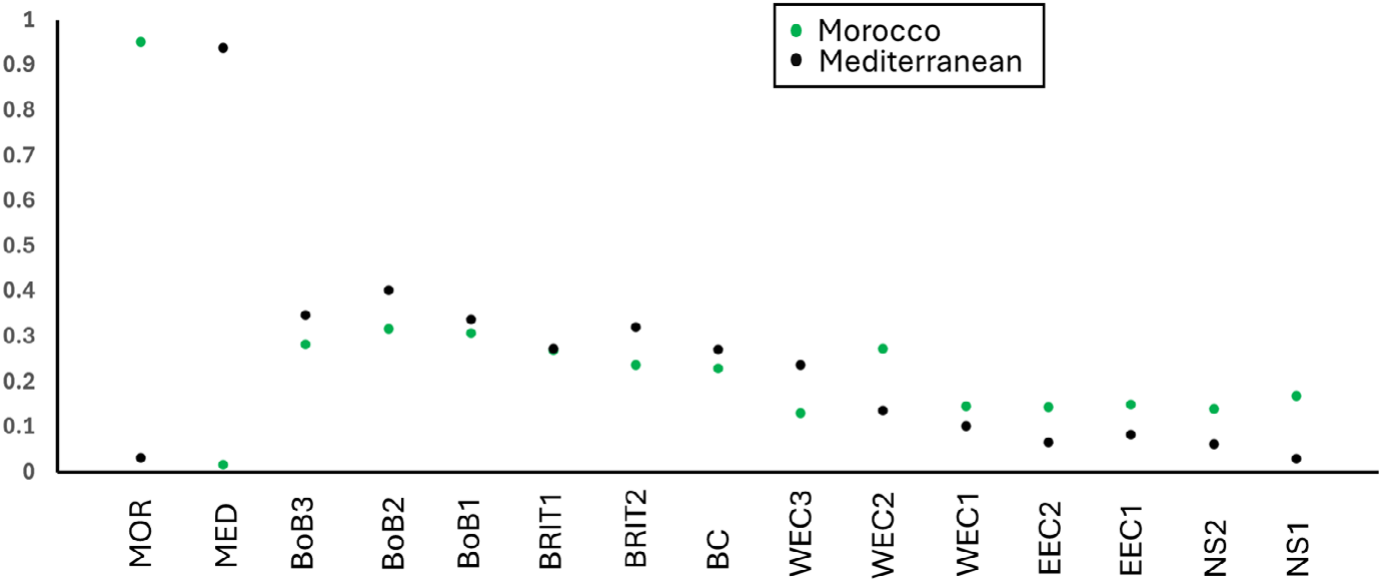
Mean Moroccan (green dots) and Mediterranean (black dots) ancestry levels inferred for each site (names correspond to Table 1) from analysis of outlier (*n*—223) loci in ADMIXTURE under a model of *K* = 3. Error bars are not shown for clarity and were highly overlapping for the NE Atlantic samples (BoB3‐NS1).

Patterns of Mediterranean and Moroccan admixture among NE Atlantic samples revealed a northward decreasing cline in both Mediterranean and Moroccan ancestry proportions (Figure 6). While there were no apparent breaks in either Moroccan or Mediterranean ancestry, there was a clear shift in relative ancestry levels at the western Channel. Specifically, levels of Mediterranean admixture were higher than Moroccan among the southern samples (Biscay to westernmost samples of the West Channel [WEC3]), but from WEC 2 to NS1, the Moroccan admixture levels were higher than Mediterranean (Figure 6).

## 4 Discussion

This study revealed three salient features. First, there was a clear separation of three regional groups: Morocco, Mediterranean Sea (represented by Gulf of Lion; GoL), and the NE Atlantic (Bay of Biscay to North Sea). Signals of non‐neutral divergence between these groups were evident at a largely overlapping suite of outlier loci identified by different tests. Second, within the NE Atlantic group, pairwise *F*_ST_ reported significant differentiation of all Bay of Biscay samples from the four North Sea and Eastern Channel samples with reduced differentiation of the geographically intermediate samples within an overall pattern of isolation by distance. Third, outlier SNPs indicated further structuring within the NE Atlantic and clearly separated a NE group (containing the North Sea and East Channel samples) from the remaining samples. This pattern indicates that the Channel may represent an area where there is an overlap of two distinct groups rather than part of a cline. The study adds to evidence of neutral (dispersal limitations) and non‐neutral (genome architecture and selection) processes shaping genetic structure of this species over different spatial and temporal scales and provides translatable information for fishery management.

### 4.1 The Outgroup Samples: Morocco and West Mediterranean

Although this study focussed on NE Atlantic sardine, the inclusion of Mediterranean and Moroccan samples allowed us to link our results with previous studies of more southern locations. The level of genetic divergence between the three groups reported here, and by da Fonseca et al. (2024) is compatible with historical glacial vicariance. Signals of differences in genetic architecture accrued during such isolation were directly revealed by da Fonseca et al. (2024) and may also explain the large number of outlier loci reported between the three groups here (Bierne et al. 2011). The differentiation between the NE Atlantic and GoL samples here aligns with the Atlantic‐Mediterranean divergence reported by Antoniou et al. (2023) and da Fonseca et al. (2024) with those studies revealing a major role for the Almeria‐Oran frontal system and historical vicariance in driving this differentiation, as seen across a host of marine species (Bargelloni et al. 2003). da Fonseca et al. (2024) included a Moroccan sample collected to the north (latitude 34.5^o^) of our Moroccan sample (latitude 23.85^o^). Interestingly, their Moroccan sardine clustered robustly with North Atlantic (Gulf of Cadiz to Brittany) sardine in what the authors labelled as the “central” group. In contrast, our Moroccan sample was highly differentiated from our NE Atlantic samples, with similar numbers of outlier and *F*_ST_ values obtained in Morocco versus NE Atlantic comparisons as in comparisons of either of these with the Mediterranean sample. As both studies included samples from the Bay of Biscay, the discordant patterns cannot be attributed to unsampled structure within the NE Atlantic. da Fonseca et al. (2024) described a highly divergent western group comprising individuals from Madeira and the Azores. Thus, there is the need for further genomic studies of sardine in the region to test if our Moroccan group corresponds to this Madeira/Azores group or is an additional lineage not sampled by da Fonseca et al. (2024). da Fonseca et al. (2024) suggest that isolation by distance, currents, and a lack of suitable habitat may serve to isolate the Madeira/Azores group from the African/European coasts, an inference supported by Kasapidis et al. (2012). Interestingly, a genomic study of anchovy has recently revealed a previously undetected lineage found in South Africa, southern Morocco, and the Canary Islands (Meyer et al. 2024). Regardless of their origin, the distinct genetic affinities of Moroccan samples revealed here and by da Fonseca et al. (2024) confirm strong population structure along the Moroccan coast. The allozyme study by Chlaida et al. (2021) proposed a robust barrier to gene flow in the Agadir Bay (30.48^o^N) that separates Moroccan sardine into North and South stocks a pattern which aligns with results from otolith microchemistry (Labonne et al. 2022). It seems likely that our Moroccan sample belongs to this Southern stock with the Moroccan sample from da Fonseca et al. (2024) to the North stock. If we also consider the retained signature of Mediterranean origin among sardine at the Canary Islands this points to deep structuring along the North African coast and a mosaic of evolutionary and ecological barriers to gene flow.

### 4.2 Clinal Genetic Structure in the NE Atlantic

Within the NE Atlantic samples, there was a significant correlation between geographical and genetic distances in both the overall dataset and the outlier dataset. Such correlations have been widely reported among small pelagic fish and attributed to spatially restricted dispersal/isolation by distance effects (Gonzalez and Zardoya 2007) that could occur at various life‐history stages in sardine. For example, larval retention mechanisms and restricted along‐shore transport have been reported for sardine (Santos et al. 2018). Similarly, adult dispersal may be limited and/or occur in a metapopulation context where population exchange rates are proportional to geographical distances (Kritzer and Sale 2004). Such processes have already been suggested by Silva et al. (2019) within the area from the northern Bay of Biscay to Gulf of Cadiz, while otolith analysis (Neves et al. 2023) also suggested that sardine move among contiguous areas.

The correlation with geographical distance was even stronger when analyzed using the outlier loci. Outlier loci may be generated by a range of non‐neutral factors such as genetic architecture and environmental selection. While the alignment between neutral and non‐neutral structure within the NE Atlantic could indicate a role for genetic architecture, this is expected to be more of a factor in contact zones between previously diverged lineages (Bierne et al. 2011). The shallow phylogeographic structure (McKeown et al. 2024) and low level of admixture among NE Atlantic samples (da Fonseca et al. 2024) suggests a role of environmental selection. In the English Channel, there is a clear double peak in spawning activity with the main periods in Spring‐early Summer and again in Autumn (Coombs et al. 2005, 2010; Stratoudakis et al. 2007). Moving to lower latitudes, the spawning activities are earlier and shorter, and Stratoudakis et al. (2007) suggested that there may be a genetic basis to upper temperature tolerance to spawning explaining such differences. Antoniou et al. (2023) reported that the number of days with sea surface temperature above 19°C (critical threshold for successful spawning sensu Stratoudakis et al. 2007) was a prominent driver of the genetic structure at neutral and non‐neutral SNPs across the Mediterranean—Cantabrian sea. Clinal variations at markers putatively under selection have also been reported in other sardine studies spanning different geographical areas (Kasapidis et al. 2012; Chlaida et al. 2006, 2009; Laurent et al. 2007). In these various cases, environmental gradients could be contributing to the observed structure via spatially varying selection (Sotka 2005, 2012) and/or local adaptation. Both these processes generate genetic differentiation but differ in the scale of gene flow and selection. In the case of spatially varying selection, the outlier patterns may be maintained despite high dispersal. The significant neutral structure between Biscay and North Sea indicates restricted dispersal and may therefore support local adaptation. Isolation by adaptation (Nosil 2009) may emerge through environmentally driven mismatches between individuals' phenotype and their environment (Marshall et al. 2010) at various life‐history stages, which in turn may serve to restrict effective gene flow by selection against migrants. While BLAST searches for some sequences containing outlier SNPs returned matches to mRNA sequences, it is challenging to draw conclusions regarding functional relationships between environment and loci. Analyses of individuals at different life‐history stages (Gagnaire et al. 2012) alongside environmental variables would enable us to better estimate the effect of environmental gradients on sardine genetic structure at different spatial scales within the NE Atlantic.

The NE Atlantic samples exhibited a northward decreasing gradient of Mediterranean and Moroccan ancestry proportions, which aligns with results from da Fonseca et al. (2024). A similar gradient in Mediterranean ancestry was reported in Atlantic seabass (Robinet et al. 2020) and used to identify cryptic dispersal barriers on the northwestern coast of the Iberian Peninsula and Brittany. Among our samples, a spike in southern group ancestry was observed for the WEC2 samples, followed by a drop in such ancestry among the higher latitude samples. While such abrupt drops can be indicative of barriers, we must take this result alone with caution given the small sample size for WEC2. There are two important considerations regarding the introgression tail analysis here. First, our sampling scheme was not optimal for such analysis (Robinet et al. 2020). Second, our outliers may not have been strong enough (introgression resistant) to provide a detectable signal (Gagnaire et al. 2015). While there was no obvious abrupt drop in admixture levels, there was a flip in relative Mediterranean and Moroccan ancestry among the West Channel samples which left the northern samples exhibiting more Moroccan than Mediterranean ancestry in contrast to the higher Mediterranean ancestry among samples of the Bay of Biscay and Bristol Channel. This could be interpreted as a signature of delayed homogenization due to spatially limited dispersal and thus implicate a barrier in that region. Such a barrier would be supported by the results of the individual assignment tests that support limited contribution of West Channel sardine to the East Channel‐North Sea. Robinet et al. (2020) reported a spike in levels of Mediterranean introgression among North Sea seabass which the authors attributed to allele surfing as part of a northward expansion rather than a local barrier effect per se. Interestingly, although da Fonseca et al. (2024) did not specifically analyze introgression tails, their population structure plots show abrupt declines in East/West group ancestry in NE Atlantic sardine around NW Iberia and Brittany. These could indicate the occurrence of localized dispersal barriers in those areas for sardine which may be obscured by gene flow (Gagnaire et al. 2015), that would align well with those reported for seabass (Robinet et al. 2020).

### 4.3 Stock Structure

McKeown et al. (2024) reported differentiation between North Sea and South Biscay sardine at a small number of outliers and mtDNA sequences. However, the overall lack of structure at “neutral” markers necessitated caution in interpretation in the context of dispersal. The larger numbers of samples and SNPs analyzed in this study provide a much more robust picture of the genetic structure of this species in this area. Pairwise *F*_ST_ values from the combined, neutral, and non‐neutral datasets revealed significant genetic differentiation in all comparisons between the Bay of Biscay and North Sea samples. Genetic differentiation between the Bay of Biscay and North Sea has been reported for multiple other species (Charrier et al. 2007; Leone et al. 2019; Huret et al. 2020) and our data confirms a level of genetic independence between the Bay of Biscay and Easter Channel/North Sea sardine populations. The outlier markers provide even more precision with individual‐based analyses emphasizing the integrity of an East Channel‐North Sea group. Specifically, individual assignment patterns indicate that while the Western Channel may be an area of mixing between putative northern and southern sardine, this mixing does not extend into the East Channel/North Sea. As sardine have the potential to spawn continuously from the Bay of Biscay to the North Sea (Huret et al. 2018; Coombs et al. 2005), and movement between the Bay of Biscay and western English Channel is supported by otolith shape analyses (Neves et al. 2021, 2023), environmental and/or behavioural factors might limit dispersal and gene flow into the East Channel‐North Sea group. The oceanography of the English Channel is rather complex with major differences in the hydrodynamics and hydrology of the western and eastern English Channel (Dauvin 2012). More specifically, the western English Channel is strongly influenced by north‐eastwards flowing Atlantic waters making the salinity and sea surface temperature conditions relatively similar to those of the Celtic sea and the Bay of Biscay. Conversely, riverine fresh‐water inflows from the Seine and Somme result in a northwards coastal current of desalinated water in the Eastern English Channel (Dauvin 2012). The eastern English Channel is also shallower than the western English Channel, exhibiting limited summer stratification in this area (Stanford and Pitcher 2004) and a higher temperature variance due to warmer summer and colder winter temperature conditions. While geographically the western and eastern Channel are separated at its narrowest point, that is, to the north of the Cotentin peninsula, hydrologically, the front separating the seasonally stratified waters in the west from well‐mixed waters in the east, is situated within the western Channel. It is a continuation of the Ushant Front (Pingree 1980), although its exact location can move seasonally and interannually. This strong structuring of the Channel may influence sardine habitat and limit gene flow, while the variable location of the boundary between its two areas may explain the imprecise genetic structure within the western Channel. The Gulf of Saint‐Malo has also been revealed as an important transition zone separating East and western English Channel benthic macrofauna (Bierne et al. 2003; Jolly et al. 2005). The analysis of samples collected at different times and life‐history stages will be needed to ascertain the likely dynamic distribution of these groups, as well as the roles and trajectories of larval/adult dispersal.

### 4.4 Management and Conservation Implications

The present study provides evidence for both large and fine scale population structure in sardine. The large‐scale differentiation between the Moroccan, West Mediterranean, Bay of Biscay, and North Sea samples confirms a high level of demographic independence among major recruitment hotspots. This regional structure is broadly consistent with current management units (ICES 2022), although further studies are needed to assess actual boundaries of the stocks particularly along the Moroccan coast. Within the NE Atlantic, we found that there was a significant differentiation between the Bay of Biscay and North Sea samples with outlier SNPs highlighting the demographic integrity of an East Channel‐North Sea group and potential overlap of groups in western English Channel. Recently, the latitudinal limit at 48° N applied for the management of Seabass between the Biscay and the North Sea stocks was questioned with both genetic (Robinet et al. 2020) and tagging studies (De Pontual et al. 2023) suggesting that the middle of the English Channel is a more appropriate boundary. Similarly for sardine, the cohesion among the Celtic Sea, West Channel, and Biscay samples is incongruent with the current Biscay and Northern stock delineation at 48° N. Sardine in the Bay of Biscay and the Channel were at one time treated as a single stock, with sardine north of 48° N only recently considered an independent stock based on evidence of differing growth rates, separate spawning grounds and the presence of all life stages in both areas (ICES 2022). Indeed, length at age is higher in the western English Channel compared to the Bay of Biscay (Menu et al. 2023). Such ecological differences and the fact that a lack of genetic differentiation can provide little information as to whether migration rates are sufficient to maintain demographic coupling (Gagnaire et al. 2015; Robinet et al. 2020) highlight the need to employ a multidisciplinary approach (e.g., combining genetics, biometrics, life‐history information, geostatistics, and oceanography) and to consider adjacent areas in the monitoring of the Biscay and West Channel sardine.

Climate change is affecting the distribution of fish populations by different mechanisms including direct displacement of populations into novel areas and increased productivity of peripheral populations (Petitgas et al. 2012; van der Kooij et al. 2024). The combined neutral and non‐neutral genetic patterns, including results of individual assignment tests, indicate that the recent increase in abundance of sardine in the North Sea results from an expansion of an East Channel‐North Sea fringe population, not an overall northward shift in the distribution; in a pattern similar to that described for anchovy in the North Sea (Petitgas et al. 2012; Huret et al. 2020). McKeown et al. (2024) reported reduced variation among a single North Sea sample collected in 2016. The results here suggest that the southern North Sea now exhibits comparable levels of variation as southern populations, though signals of leading‐edge drift may be apparent in the proportions of southern group ancestry (Robinet et al. 2020). While this demonstrates the ability of these peripheral populations to track changes in their habitat, the mosaic of neutral and non‐neutral structuring revealed here and by other studies (Antoniou et al. 2023; da Fonseca et al. 2024) raises questions as to how southern populations may respond to climate changes at the leading edge of their distributions. Integrating genome wide data across the species' range will be important to understand the factors shaping the resilience of sardine populations to future climate conditions and harvesting pressure.

## Conflicts of Interest

The authors declare no conflicts of interest.

## Supporting information


Figure S1.

Table S1.

Table S2.

## Data Availability

All data for this study are available from the open access repository at https://pure.aber.ac.uk/.
