# Supplementary material for: Hierarchical Neutral and Non‐Neutral Spatial Genetic Structuring in the European Sardine (Sardina pilchardus) Revealed by Genomic Analysis: Implications for Management
Source: Evol Appl. 2025 Apr 1;18(4):e70080. doi: 10.1111/eva.70080 (PMC11961398; doi:10.1111/eva.70080)
Supplement: Supplementary file 1 — Figure S1. [file EVA-18-e70080-s002.docx]

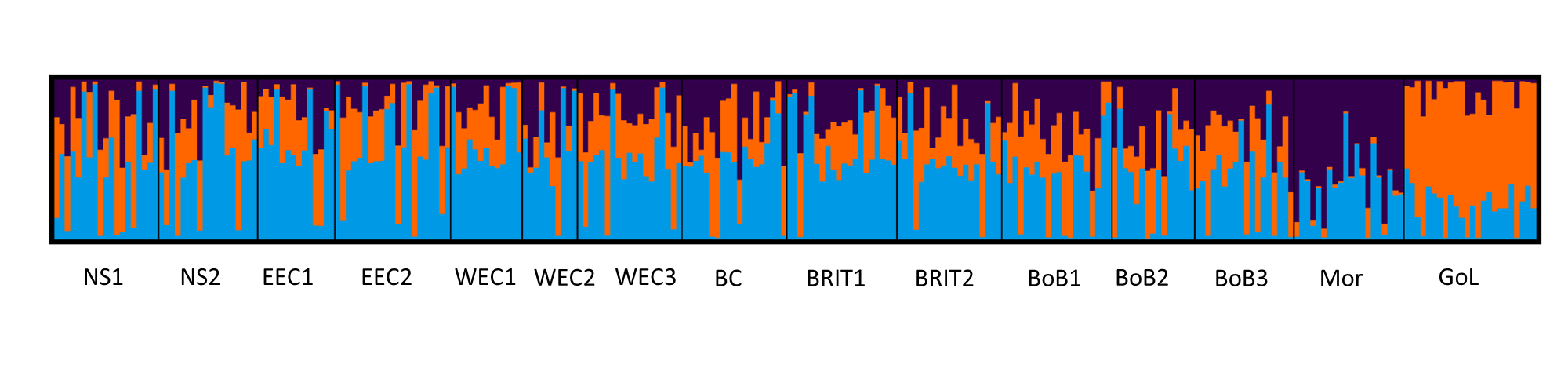


Supplementary Figure 1. Barplot showing clustering results under the optimal model of K = 3 for each individual based on 3369 presumed ‘neutral’ loci i.e. total SNPs (n =3592) minus outlier SNPs (n = 223).
