# Supplementary material for: Hierarchical Neutral and Non‐Neutral Spatial Genetic Structuring in the European Sardine (Sardina pilchardus) Revealed by Genomic Analysis: Implications for Management
Source: Evol Appl. 2025 Apr 1;18(4):e70080. doi: 10.1111/eva.70080 (PMC11961398; doi:10.1111/eva.70080)
Supplement: Supplementary file 2 — Table S1. [file EVA-18-e70080-s001.docx]

Supplementary Table 1. Overview of the number of outliers detected by the FDIST and BAYESCAN methods for the different combinations of samples.

| Sample configuration | FDIST | BAYESCAN |
| --- | --- | --- |
| Mediterranean & NE Atlantic | 229 | 119 |
| Morocco & NE Atlantic | 421 | 173 |
| Morocco & Mediterranean | 672 | 111 |
