# Supplementary material for: Hierarchical Neutral and Non‐Neutral Spatial Genetic Structuring in the European Sardine (Sardina pilchardus) Revealed by Genomic Analysis: Implications for Management
Source: Evol Appl. 2025 Apr 1;18(4):e70080. doi: 10.1111/eva.70080 (PMC11961398; doi:10.1111/eva.70080)
Supplement: Supplementary file 3 — Table S2. [file EVA-18-e70080-s003.docx]

Supplementary Table 2. Summary information for the subset of outlier SNPs that revealed significant similarity to mRNA sequences according to BLAST.

| Outlier SNP | BLAST hit | % identity | NCBI accession |
| --- | --- | --- | --- |
| Sardine_1043809 | *Sardina pilchardus* LIM domain transcription factor LMO4.1 (si:dkey-90l8.3), mRNA | 97 | XM_062543192.1 |
| Sardine_1058637 | *Sardina pilchardus* leucine-rich repeat transmembrane neuronal protein 4 (LOC134089344), partial mRNA | 99 | XM_062543785.1 |
| Sardine_111072 | *Sardina pilchardus* FKBP prolyl isomerase 5 (fkbp5), mRNA | 99 | XM_062546193.1 |
| Sardine_2021_1183310 | *Sardina pilchardus* GDNF family receptor alpha like (gfral), transcript variant X2, mRNA | 99 | XM_062519864.1 |
| Sardine_2021_1217291 | *Sardina pilchardus* leukotriene B4 receptor 1-like (LOC134095286), mRNA | 99 | XM_062548735.1 |
| Sardine_2021_1228393 | *Alosa alosa* kyphoscoliosis peptidase-like (LOC125311018), transcript variant X2, mRNA | 92 | XM_048268821.1 |
| Sardine_2021_1311408 | *Sardina pilchardus* asparaginase homolog (S. cerevisiae) (aspg), transcript variant X2, mRNA | 99 | XM_062536438.1 |
| Sardine_2021_146817 | *Sardina pilchardus* inositol-trisphosphate 3-kinase B (si:ch73-22a13.3), mRNA | 100 | XM_062549187.1 |
| Sardine_2021_173850 | *Alosa alosa* tenascin Ca (tnca), mRNA | 99 | XM_048258146.1 |
| Sardine_2021_203372 | *Sardina pilchardus* dolichol kinase (dolk), transcript variant X3, mRNA | 100 | XM_062554010.1 |
| Sardine_2021_233892 | *Sardina pilchardus* RanBP-type and C3HC4-type zinc finger containing 1 (rbck1), transcript variant X2, mRNA | 97 | XM_062545976.1 |
| Sardine_2021_262568 | *Sardina pilchardus* FKBP prolyl isomerase 5 (fkbp5), mRNA | 97 | XM_062546193.1 |
| Sardine_2021_264588 | *Sardina pilchardus* klotho (kl), mRNA | 97 | XM_062537054.1 |
| Sardine_2021_277741 | *Sardina pilchardus* zinc finger protein 154-like (LOC134088502), transcript variant X3, mRNA | 93 | XM_062542483.1 |
| Sardine_2021_319337 | *Sardina pilchardus* cadherin 15, type 1, M-cadherin (myotubule) (cdh15), mRNA | 100 | XM_062549500.1 |
| Sardine_2021_326373 | *Sardina pilchardus* enhancer of mRNA-decapping protein 3-like (LOC134096256), transcript variant X6, mRNA | 98 | XM_062550031.1 |
| Sardine_2021_355856 | *Sardina pilchardus* ankyrin repeat domain 11 (ankrd11), mRNA | 99 | XM_062550014.1 |
| Sardine_2021_41834 | *Sardina pilchardus* uncharacterized LOC134062240 (LOC134062240), mRNA | 99 | XM_062518193.1 |
| Sardine_2021_443994 | *Sardina pilchardus* zinc finger and BTB domain containing 20 (zbtb20), mRNA | 99 | XM_062537159.1 |
| Sardine_2021_456673 | *Sardina pilchardus* gap junction Cx32.2 protein-like (LOC134098119), mRNA | 99 | XM_062551082.1 |
| Sardine_2021_555812 | *Sardina pilchardus* collagen alpha-1(XXVII) chain A-like (LOC134100382), mRNA | 99 | XM_062553559.1 |
| Sardine_2021_559874 | *Sardina pilchardus* SH3 domain-containing kinase-binding protein 1 (si:dkey-71d15.2), transcript variant X2, mRNA | 100 | XM_062547782.1 |
| Sardine_2021_570923 | *Alosa sapidissima* glutaminyl-peptide cyclotransferase-like b (qpctlb), mRNA | 86 | XM_042093791.1 |
| Sardine_2021_625073 | *Sardina pilchardus* aggrecan core protein-like (LOC134095298), mRNA | 99 | XM_062548747.1 |
| Sardine_2021_699373 | *Sardina pilchardus* serine/threonine-protein kinase 38-like (LOC134062161), mRNA | 100 | XM_062518079.1 |
| Sardine_2021_733466 | *Sardina pilchardus* toll-like receptor 8 (LOC134078587), transcript variant X2, mRNA | 98 | XM_062534615.1 |
| Sardine_2021_764208 | *Sardina pilchardus* lactase/phlorizin hydrolase-like (LOC134077968), mRNA | 98 | XM_062533601.1 |
| Sardine_2021_76563 | *Sardina pilchardus* cystein proteinase inhibitor protein salarin-like (LOC134067253), transcript variant X2, mRNA | 91 | XM_062522415.1 |
| Sardine_2021_911334 | *Sardina pilchardus* prospero homeobox protein 1-like (LOC134097275), transcript variant X3, mRNA | 100 | XM_062550125.1 |
| Sardine_2021_929287 | *Sardina pilchardus* growth arrest-specific protein 2 (LOC134093500), mRNA | 95 | XM_062546556.1 |
| Sardine_2021_946574 | *Sardina pilchardus* sema domain, immunoglobulin domain (Ig), transmembrane domain (TM) and short cytoplasmic domain, (semaphorin) 4Ba (sema4ba), transcript variant X4, mRNA | 97 | XM_062546582.1 |
| Sardine_2021_954652 | *Sardina pilchardus* zinc finger protein 154-like (LOC134088502), transcript variant X3, mRNA | 97 | XM_062542483.1 |
| Sardine_2021_969950 | *Sardina pilchardus* polymerase (DNA directed) kappa (polk), mRNA | 99 | XM_062543230.1 |
| Sardine_2021_986797 | *Sardina pilchardus* MPN domain containing (mpnd), mRNA | 97 | XM_062556811.1 |
